# Nanofibers are a matter of perspective: effects of methodology and subjectivity on diameter measurements†

**DOI:** 10.1039/d3na00528c

**Published:** 2023-10-11

**Authors:** Martin Wortmann, Michael Westphal, Bernhard Kaltschmidt, Michaela Klöcker, Ashley S. Layland, Bennet Brockhagen, Andreas Hütten, Natalie Frese, Andrea Ehrmann

**Affiliations:** a Bielefeld University, Faculty of Physics, Universitätsstraße 25 33615 Bielefeld Germany mwortmann@physik.uni-bielefeld.de; b Bielefeld University of Applied Sciences and Arts, Faculty of Engineering and Mathematics Interaktion 1 33619 Bielefeld Germany; c neotem Bioanalytics, Universitätsstraße 25 33615 Bielefeld Germany; d University of Hawaii, Department of Physics and Astronomy Watanabe Hall, 2505 Correa Road Honolulu HI 96822 USA

## Abstract

Nanofibers are currently among the most researched nanomaterials in materials science. Various high-resolution microscopy techniques are used for morphological investigations, with the diameter as primary characteristic. Since methodological factors influencing the diameter distribution are usually ignored, numerical values can hardly be compared across different or even within single studies. Here, we investigate influencing factors such as microscopy technique, degree of magnification, eventual coatings, and the analysts' bias in the image selection and evaluation. We imaged a single nanofiber sample using scanning electron microscopy (SEM), helium ion microscopy (HIM), atomic force microscopy (AFM), and transmission electron microscopy (TEM). These techniques yield significant methodological variations between the diameter distributions. We further observed a strong influence of analysts' subjectivity, with a consistent average deviation between 4 different analysts of up to 31%. The average deviation between micrographs within each category was 14%, revealing a considerable influence of micrograph selection and strong potential for cherry picking. The mean values were mostly comparable with the results using automated image analysis software, which was more reproducible, much faster, and more accurate for images with lower magnification. The results demonstrate that one of the most frequently measured characteristics of nanofibers is subject to strong systematic fluctuations that are rarely if ever addressed.

## Abstract 2

The measurement of nanofiber diameters from micrographs is highly dependent on methodology and the analyst's subjective evaluation.

## Graphical abstract

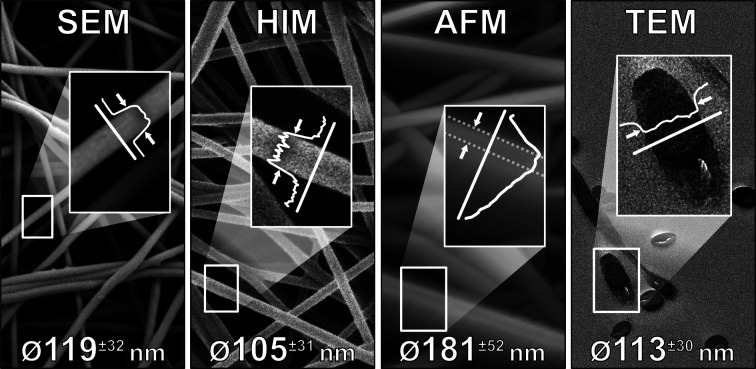

## 1. Introduction

For decades, nanofibers have been studied in numerous fields of research, either as naturally occurring building blocks or as synthetic materials for a wide range of applications. Since the advent of electrospinning, polymeric nanofibers, in particular, have become one of the most studied nanomaterials in materials science. Electrospinning enables the production of nanofibers from diverse polymers or polymer blends, optionally incorporating active ingredients, nanoparticles, and other additives. Their properties can be controlled in a wide range by the spinning solution and process parameters.^1–3^ With their exceptional structural properties and unique surface characteristics, nanofibers exhibit immense potential for diverse applications, including electronics, energy storage, filtration, tissue engineering, and environmental remediation, to name just a few.^4,5^

Depending on the intended application, a variety of characteristics are typically investigated, such as the diameter, orientation, porosity, specific surface area, mat thickness, mechanical properties, electrical and magnetic properties, or permeability for gases or liquids.^6^ High-resolution microscopy is the primary method for nanofiber characterization, and most publications in this field report diameter distributions based on micrographs. The mean diameter is usually the most important morphological parameter, and is often correlated with mechanical or other physicochemical properties that determine their applicability.^7–11^ The diameter can be well controlled by electrospinning *via* various process parameter;^12,13^ however, even when determining such correlations, systematic methodological influences on the diameter measurement are usually neglected. Although exact figures are not always of primary interest, many studies have reported correlations between material properties, environmental parameters, spinning solution, process parameters and the resulting fiber diameters.^7,12,14^ Owing to the sheer number of studies in which the diameter distribution is assessed, a better understanding of the systematic errors and influencing factors is of utmost importance.

Scanning electron microscopy (SEM) is the workhorse of material science. It is widely accessible and thus by far the most often applied technique for the morphological analysis of nanofibers. Since it is fast, easy to use, and can image large amounts of nanofibers with little sample preparation and high resolution, it is also most commonly used to determine diameter distributions. Transmission electron microscopy (TEM) is also widely used and excels in its ability to visualize the fiber cross-section at up to atomic resolution. Helium ion microscopy (HIM) is in many regards similar to SEM, however, hitherto much less established and accessible. It has already been used in several studies to image nanofibers^8,15,16^ and it is of particular interest for diameter measurements due to its capacity to image insulating samples without prior coating.^17^ Atomic force microscopy (AFM) is often used to characterize nanofiber properties beyond mere morphology, such as magnetization, friction, or mechanical strength.^18–21^ The micrographs are commonly analyzed manually using image analysis software to determine diameter distributions. Some automated image analysis tools have been developed that allow for rapid batch analysis, *e.g.* General Image Fiber Tool (GIFT), DiameterJ, or BoneJ, to name just a few. Such tools promise apparently unbiased and reproducible fiber diameter assessments.^22–28^

Here, we discuss the results of diameter measurements performed on a single electrospun nanofiber nonwoven using different microscopic methods, *i.e.* SEM, HIM, TEM, and AFM. The results reveal significant deviations in the diameter distribution depending on the imaging technique, imaged sample region, and subjectivity of the analysts. Deviations were also found between human analysts and automated image analysis using the GIFT and DiameterJ software implemented in ImageJ. These factors are rarely if ever addressed, making published correlations between material properties and nanofiber diameters highly unreliable and difficult to reproduce, which may inadvertently contribute more broadly to the so-called reproducibility crisis in the field of materials science.^29,30^

## 2. Experimental

Nanofibers were electrospun from a polymer solution of 16 wt% PAN (XPAN, Dralon, Germany) dissolved in dimethyl sulfoxide (DMSO, min 99.9%, S3 chemicals, Germany) using a wire-based spinning device Nanospider Lab (Elmarco Ltd., Liberec, Czech Republic). A sample of 5 mm × 5 mm was cut from the as produced nonwoven. One half of it was coated with gold with a nominal thickness of 10 nm using magnetron sputtering; the other half was covered during gold deposition and thus left unmodified. This sample was then investigated using SEM, HIM, and AFM:

SEM images were taken with a Sigma 300 VP SEM (Carl Zeiss Microscopy GmbH, Oberkochen, Germany) with an acceleration voltage of 7 kV. 10 micrographs with 4.8 μm × 6.4 μm field of view (FOV) and 1 with 15.4 μm × 20.5 μm FOV were taken of the coated sample half each.

HIM images were taken with a HIM Orion Plus (Carl Zeiss, Jena, Germany) using an acceleration voltage of 33.5 kV, a beam current of 0.2 pA and spot control of 7. The pristine fibers were imaged using an electron flood gun for charge compensation. 10 micrographs with 3 μm × 3 μm FOV and 1 with 20 μm × 20 μm FOV were taken of the pristine and coated sample half each.

**Fig. 1:**
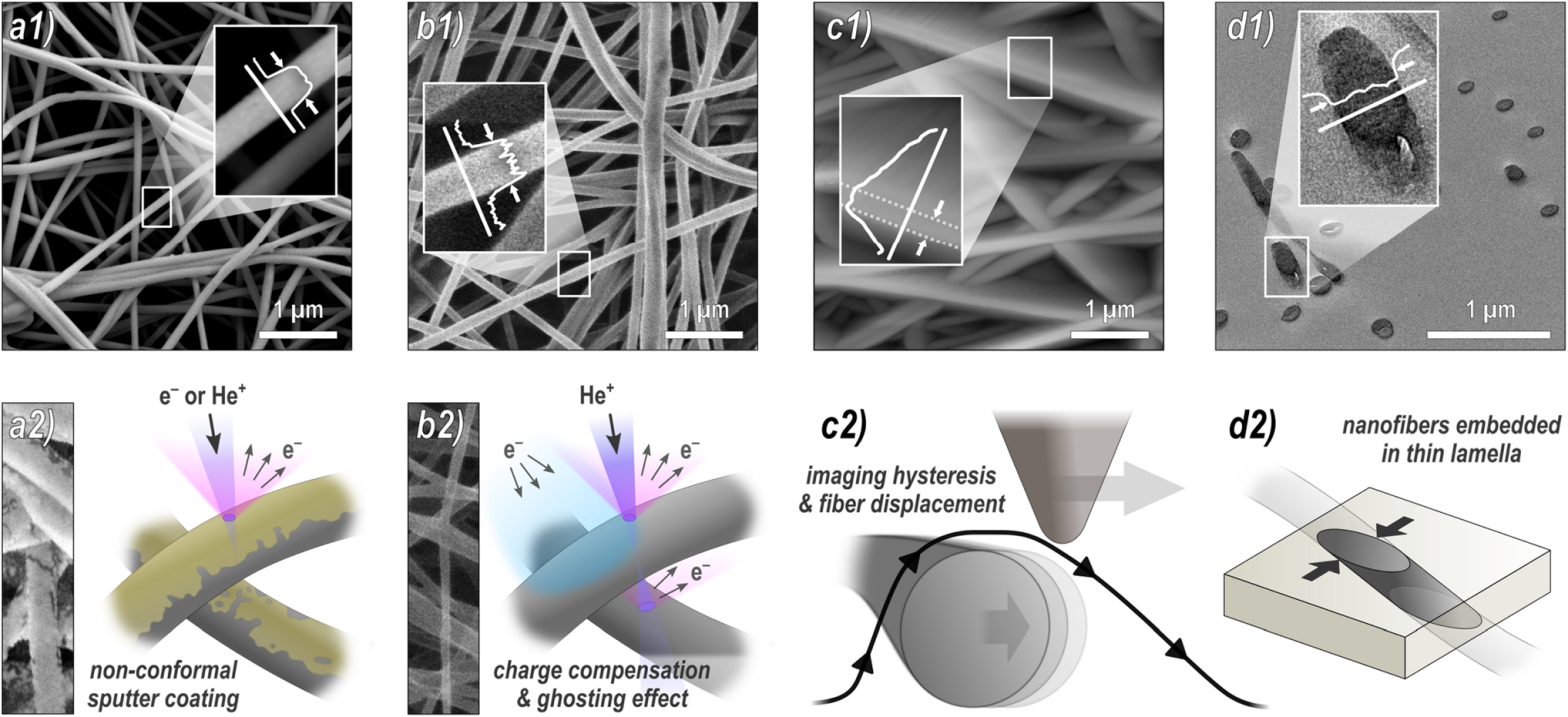
Overview of some common microscopy techniques for nanofiber characterization: (a) SEM, (b) HIM, (c) AFM, and (d) TEM. On the top are exemplary micrographs with inserted grey-scale plots (arrows indicate where the diameter was measured) and on the bottom are schematic illustrations of the respective technique's working principles.

AFM micrographs were taken in a FlexAFM Axiom (Nanosurf, Liestal, Switzerland) in tapping mode with a TAP190Al-G cantilever (tip radius 10 nm, half cone angle 10° at the apex), which was regularly renewed. The FOV was 5 μm × 5 μm. The standard settings were: 512 points per line, scan rate 1.4 s per line, set point 60%, P-gain 550, I-gain 1000, D-gain 0, free vibration amplitude 4–6 V, depending on the surface roughness. It should be mentioned that these free vibration amplitude values are necessarily much higher than in case of very flat surfaces, leading to less sharp images and stronger tracking effects, as depicted in Fig. 1c.

For TEM imaging, an additional sample of 1 mm × 1 mm was cut from the initial nonwoven, inserted in a 1 : 1 mixture of acetone and epoxy resin (TAAB Laboratories Equipment Ltd, UK), then incubated at room temperature for 20 min, followed by infiltration in a desiccator for 1 h. After curing of the resin at 70 °C for 12 h, ultra-thin lamellae of about 70 nm was prepared using an ultramicrotome (Ultracut, Reichert-Jung, Austria). The lamella was then contrasted for 5 min by lead citrate (Plano GmbH, Germany) and uranyl acetate (Science Services GmbH, Germany). TEM images were taking using a field emission electron microscope JEOL JEM-2200FS (JEOL Ltd., Japan) operating at 200 kV. A total of 20 micrographs with 3 μm × 3 μm FOV and 1 micrograph of 26.7 μm × 26.7 μm FOV were examined.

All sample regions to be imaged were chosen arbitrarily by the operators without instructions or suggestions regarding an expected outcome. However, areas with major morphological deviations such as large agglomerates or membranous areas were intentionally excluded during the imaging. The following numbers of nanofibers have been measured in parallel by 4 different analysts using the ImageJ software: HIM and AFM of coated nanofibers – 10 fibers in 10 images each; HIM and AFM of pristine nanofibers – 10 fibers in 10 images each; SEM – 10 fibers in 10 images each for higher magnification and 30 fibers in 1 image with lower magnification; TEM – 5 fibers in 20 images each for higher magnification and 30 fibers in 1 image with lower magnification. The analysts evaluated the fiber diameters by marking two opposite edges of a fiber either by visual judgment directly in the image or in the grey scale plot, with the connection line perpendicular to the fiber axis, as depicted in Fig. 1. Although there are methods for manual image analysis that assure more precise measurements, those are rarely used or at least virtually never disclosed in publications. Thus, no strict instructions were prescribed. Due to the rapid greyscale change between a fiber and its environment (*cf.*Fig. 1), there was no advantage of applying line scans and measuring at the full-width half-maximum (FWHM) or using similar image processing techniques. It should be mentioned that the minimum uncertainty is of the order of one pixel, which corresponds to 3.8 nm (high mag. SEM), 10 nm (low mag. SEM), 4.9 nm (high mag. HIM), 19.3 nm (low mag. HIM), 10 nm (AFM), 0.73 nm (high mag. TEM), and 6.5 nm (low mag. TEM).

In addition, the images have been analyzed using GIFT and DiameterJ, which are implemented in ImageJ, here, using version 1.54f and 1.51w, respectively. The underlying algorithms have been described in detail elsewhere.^24,31,32^ GIFT automatically fits a Gaussian peak function to the primary peak in the generated diameter histogram. For the batch analysis of the highly magnified SEM and HIM images (10 and 5, respectively), the raw data of each individual image has been combined and fitted by a Gaussian function in the OriginPro 2023 software. It should be mentioned that the amount of generated data points can vary significantly between different micrographs, which is why some images might be overrepresented in the combined data set. An alternative approach would be to calculate the mean and STD of the individual image results. DiameterJ combines the data sets from batch analysis automatically and calculates the cumulative average value as a result. The Gaussian fits were again calculated using OriginPro 2023. DiameterJ uses 3 different segmentation algorithms, each generating 8 segmented images. Based on visual judgement of the operator, the most accurate segmentation is chosen for further processing. The results can vary depending on this subjective choice.

## 3. Results & discussion

To investigate the influence of the methodology on the diameter distribution, we used four different microscopy techniques to take multiple images of the same nanofiber sample, which were independently analyzed by four different analysts (all micrographs can be found in the ESI†). The methodology of the analysis was discussed in advance to ensure that any differences were due to the subjective selection of nanofibers included in the analysis.

Fig. 1 provides an overview of the techniques used: (a) SEM is by far the most frequently used method. To avoid charging of the sample during imaging, it is typically sputter-coated with a nanometer-thin conductive material such as gold, which has a potential influence on the diameter measurement. (b) HIM is much less common, but has some advantages over SEM. Its working principle is very similar to that of SEM; however, it uses He^+^ ions instead of electrons as the primary particle beam. In contrast to the SEM, this causes positive surface charging on insulation materials such as pristine, uncoated nanofibers, which can be compensated by an electron flood gun without compromising the fibers by a sputter coating.^33,34^ Here, we acquired HIM images from both pristine and coated nanofibers. Although the theoretical resolution of HIM is higher than that of SEM, imaging of freestanding, insulating, and highly ion-penetrable materials is much more challenging and time consuming, which is why the resolution is lower compared to SEM in this case. (c) AFM is often used because it provides more information than mere imaging, such as topographical, mechanical, electrical, or magnetic properties.^20,35^ Again, both pristine and coated fibers were imaged by AFM. As we will see, it is, however, the least reliable method for diameter measurements. (d) TEM is probably the second most frequently used technique for nanofiber characterization. Its greatest strength compared to the other methods is the imaging of fiber cross-sections with very high resolution, revealing structural heterogeneity within the nanofibers up to atomic resolution.^36^ For sample preparation, nanofibers are usually embedded in resin, followed by thin sectioning by ultramicrotomy. An alternative method, which is, due to its simplicity, frequently used for sample preparation, is to disperse the nanofibers in a liquid and then deposit them directly onto a grid. This method visualized the fibers from the side, similar to SEM images. It has not been examined in this study.

**Fig. 2:**
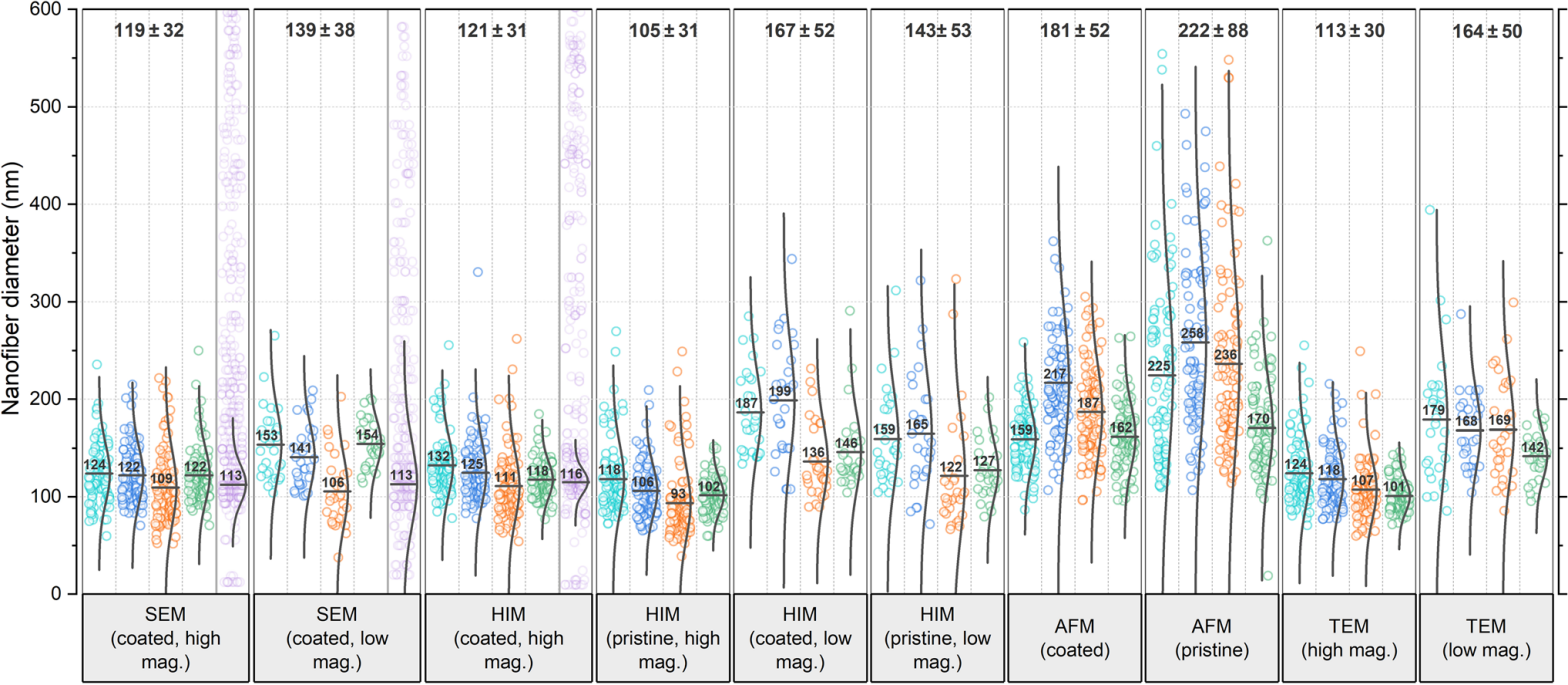
Comparison of nanofiber diameters obtained from various microscopy techniques and analyzed by 4 different analysts each, referred to as A1 (teal), A2 (blue), A3 (orange), and A4 (green). Data points shown in purple have been generated by the GIFT macro in the ImageJ software (the number of generated data points has been drastically reduced in the figure for better visibility). These data are shown again as a histogram in comparison with data generated by DiameterJ in Fig. S7 in the ESI.† In the software-generated data sets, the Gaussian distribution plot has been fitted to the primary peak of the histogram, whereas for the analyst's data sets, the plots are calculated for all data points. All numerical values were rounded to whole numbers. The total mean and STD values shown on top of the diagram do not contain the software-generated data.

The comparison reveals several systematic variables in the analysis of nanofiber diameters from micrographs, as depicted in Fig. 2: SEM and HIM of gold sputter-coated nanofibers are in good agreement. As illustrated in Fig. 1a, nonconformal conductive coatings for electron microscopy are typically produced by sputter deposition. They are affected by clustering (particularly on organic substrates^37^) and shadow casting, which may distort the appearance of the fiber. The HIM images showed a difference in mean diameter between pristine and gold-coated nanofibers of 16 and 24 nm with the same standard deviations (STD) at high and low magnification, respectively, which could be attributed to the coating (nominal thickness 10 nm). The nanofibers imaged by TEM were not coated. Their mean diameter was also lower than in SEM, but slightly higher than in HIM. Possibly the fiber cross-sections appear slightly thicker as a result of the absorption of resin components during embedding or shearing during ultramicrotomy. It is unclear whether this effect is of methodological origin or due to mere chance. It can be assumed that the results of charge-compensated HIM imaging are closest to the true diameters, as there is apparently no systematic distortion. The apparent thickness should not be affected by the charge compensation and ghosting effect, which results from the transmission of He^+^ ions and emission of secondary electrons from the underlying fibers,^38^ as illustrated in Fig. 1b.

In all categories, the analysts reported significantly higher mean values and STD for low mag. images than for high mag. images: the increase in mean value from low to high mag. was 17% for SEM, 38% for HIM coated, 36% for HIM pristine, and 45% for TEM. A less accurate evaluation with a higher STD was to be expected because of the lower resolution/pixel width (see Experimental section). However, with a sufficient number of measurements, this should not affect the mean value. Identical fibers do not appear thicker at lower magnification, which was confirmed by imaging the same sample region at different levels of magnification (these results are shown in Fig. S6 in the ESI†). Therefore, the fact that larger mean values were measured in all categories can be attributed only to subjective preferences in the selection of nanofibers. It is plausible that larger fibers were measured preferentially because of their better visibility; smaller fibers might have even been barely noticeable.

Analysis of the AFM images shows by far the largest average values and STD, approximately twice as large as the values obtained from HIM images of uncoated fibers. There are two major reasons for this apparent increase in diameter: (1) the radius of the AFM tip broadens the apparent diameter^39^ (here, we expect about twice the tip radius of 10 nm) and (2) the individual or collective elastic displacement of fibers in scan direction depending on morphological characteristics, such as nonwoven density and orientation. These effects lead to a smudged appearance of the fibers, which makes an accurate evaluation difficult, causing a large STD and a strong influence of subjectivity in the evaluation of micrographs. When comparing the forward and backward scan directions, a clear hysteresis effect can be seen in the fibers that are not parallel to the scan direction, which, as shown in Fig. 1c, can affect the measured diameter. An exemplary difference image between forward and backward scan direction is shown in Fig. S5 in the ESI.† This tracking effect is influenced by set point, free vibration amplitude, scan rate, and gains. The difference between the gold-coated and pristine fibers can be attributed to the lower rigidity of the pristine fibers, which increased the average displacement in scan direction. As the mobility varies across different positions within the nonwoven, this is accompanied by an elevated STD with very large outliers. This also manifested in a comparably large number of failed imaging attempts for the pristine fibers. Based on these results, more accurate measurements are expected when a metallic coating is applied prior to AFM imaging.

It is striking that the relative results of the different analysts A1–A4 for each microscopy method are very similar. For instance, A1 reported the highest mean value in most categories. A3 and A4 measured the lowest mean values in almost all categories. A2 measured larger diameters than A3 in every category (average difference 17%), implying a strong subjective factor in the methodology or choice of nanofibers to include in the analysis. The average deviation for all categories between lowest and largest reported mean value is a remarkable 31%. The average STD of mean values reported by the analysts within each category is 19%, which can be interpreted as the variation to be expected solely based on the choice of analyst.

In the literature, the diameter distribution is usually determined by one analyst from a single micrograph, so that the mere choice of the imaged sample region can substantially influence both qualitative and quantitative results, which is even more problematic, when considering the scientist's expectations and confirmation bias. The mean values per micrograph vary with an average STD of ±22 nm averaged over all categories, which is an average of 14% of the mean value within each category. Such strong variations between micrographs even on a small sample area and within common categories pose the risk of misinterpretation of apparent correlations along with great potential for cherry-picking.^30^

A variety of automated image analysis tools are available. The algorithms underlying the measurement process may differ, however, a common approach is to fit a Gaussian function in the primary peak of the diameter histogram to dispose of apparently faulty measurements. Here, we use GIFT as well as DiameterJ, both implemented in ImageJ, for a comparison with the manual measurements. As these tools were developed for SEM images (like most comparable software), reasonable data could only be obtained from the SEM images and some of the high magnification HIM images of coated fibers. In most HIM images and all AFM images the fiber edges were too blurred for both algorithms to provide reliable data. In such cases, GIFT produced random diameter distributions with no clear peak in the histogram, whereas DiameterJ's results became highly dependent on the subjective choice of segmented images, often resulting in distorted distributions insufficient for an accurate analysis. GIFT's results are shown in purple in Fig. 2 and DiameterJ's results are shown for comparison in Fig. S7 in the ESI.† Some studies compared the results of automated *versus* human diameter measurements (usually to validate the former) and mostly found good agreement, often emphasizing significant advantages in labor intensity and reproducibility.^22,24,32,40^ Such studies usually focus on over-ideal cases of high resolution, high contrast SEM images of monodisperse, narrowly distributed fiber diameters on a discernible clear background. Non-Gaussian diameter distributions and suboptimal micrograph resolution are, however, likely to introduce systematic errors, as real diameter measurements deviating from the normal distribution are indistinguishable from noise. Most of the analysts' results as well as the software-generated results show slightly asymmetric distributions with data spread out more towards larger diameters. It is unsurprising then, that the mean values obtained from GIFT and DiameterJ are slightly smaller than the analysts', as larger values are discarded as noise. Using a log-normal distribution instead of the conventional Gaussian distribution might actually be a more accurate approach in general. Interestingly, in contrast to the analysts, both software tools gave the same mean value for SEM images with high and low magnification, again confirming that the higher mean value for low magnification measured by the analysts is due to unconscious selection bias, which may be circumvented by automated analysis software.

## 4. Conclusion

Depending on the microscopy technique, a variety of factors can lead to an apparent increase in nanofiber diameter, with charge-compensated HIM of uncoated fibers likely to yield the most accurate values, and AFM of uncoated fibers is likely to yield the least accurate values. It was found that different analysts consistently reported different results across all tested categories and tended to measure thicker fibers at lower magnifications, which led to significant overestimation of the diameter. Significant variations within the categories have been observed both between different micrographs (14% STD of the mean value) and between different analysts (19% STD of the mean value). Automated image analysis software, although relatively scarcely used, is reproducible and much faster than manual measurements. The mean values were slightly lower, likely discarding larger diameters as noise, but the results were mostly comparable with the analysts' at high magnification. In contrast to the analysts, the software gave the same mean values at high and low magnification. However, meaningful results were only obtained for SEM images and few HIM images owing to the software's demands on image type and quality.

When using numerical values, it is important to consider the strong influence of methodology and subjectivity, and possibly bias, in determining the fiber diameter distributions. If systematic influencing factors are not taken into account, it can be assumed that many published correlations between fiber diameter and material properties are significantly less reliable and more difficult to reproduce than they appear based merely on their standard deviation.

## Author contributions

M. K. and M. Wo. made SEM images; N. F. and M. We. made HIM images; A. E. made AFM images; B. K. made TEM images; A. L. prepared the samples for TEM; A. E., B. B., M. We., and M. Wo. evaluated the images; A. E., A. H., N. F., and M. Wo. planned and supervised the experimental work; M. Wo. and A. E. wrote the manuscript with the assistance of all co-authors; A. E., A. H., and N. F. revised the manuscript.

## Conflicts of interest

The authors declare no conflict of interest.

## Supplementary Material

NA-005-D3NA00528C-s001
