# Supplementary material for: Nanofibers are a matter of perspective: effects of methodology and subjectivity on diameter measurements
Source: Nanoscale Adv. 2023 Oct 11;5(21):5900–6. doi: 10.1039/d3na00528c (PMC10597546; doi:10.1039/d3na00528c)
Supplement: NA-005-D3NA00528C-s001 [file NA-005-D3NA00528C-s001.pdf]

Electronic Supplementary Material (ESI) for Nanoscale Advances.
This journal is © The Royal Society of Chemistry 2023

Supporting Information

Nanofibers are a Matter of Perspective: Effects of Methodology and Subjectivity on Diameter Measurements

Martin Wortmann[1,*], Michael Westphal[1], Bernhardt Kaltschmidt[1], Michaela Klöcker[2], Ashley S. Layland[3], Bennet Brockhagen[2], Andreas Hütten[1], Natalie Frese[4], Andrea Ehrmann[2]

[1] Bielefeld University, Faculty of Physics, Universitätsstraße 25, 33615 Bielefeld, Germany
[2] Bielefeld University of Applied Sciences and Arts, Faculty of Engineering and Mathematics, Interaktion 1, 33619 Bielefeld, Germany
[3] neotem Bioanalytics, Universitätsstraße 25, 33615 Bielefeld, Germany
[4] University of Hawaii, Department of Physics and Astronomy, Watanabe Hall, 2505 Correa Road, Honolulu, HI 96822, USA
***** Correspondence: mwortmann@physik.uni-bielefeld.de

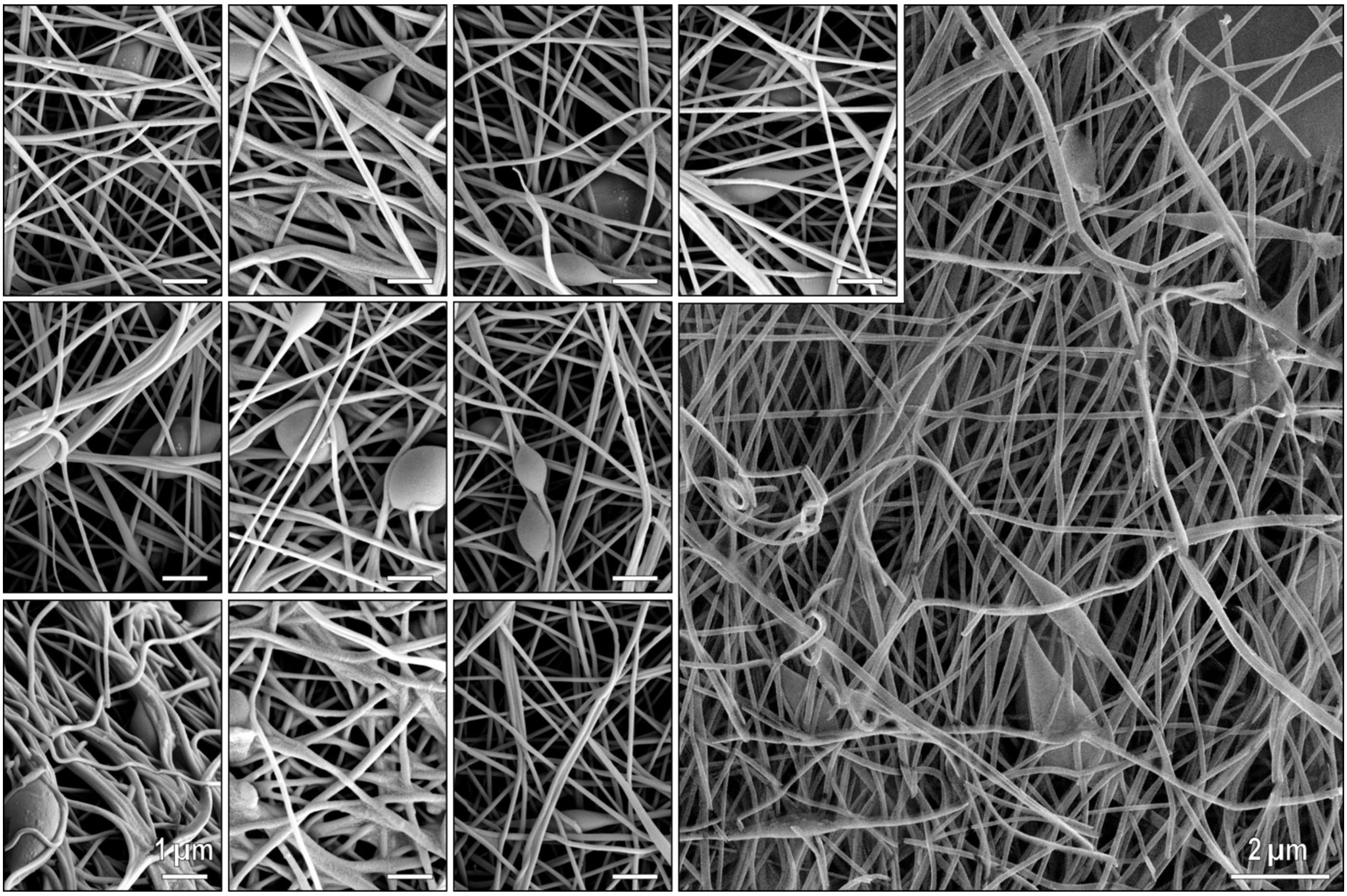

Figure S1: All SEM images that have been analyzed. The images on the left are referred to as *high mag*. and the larger one on the right is referred to as *low mag*.

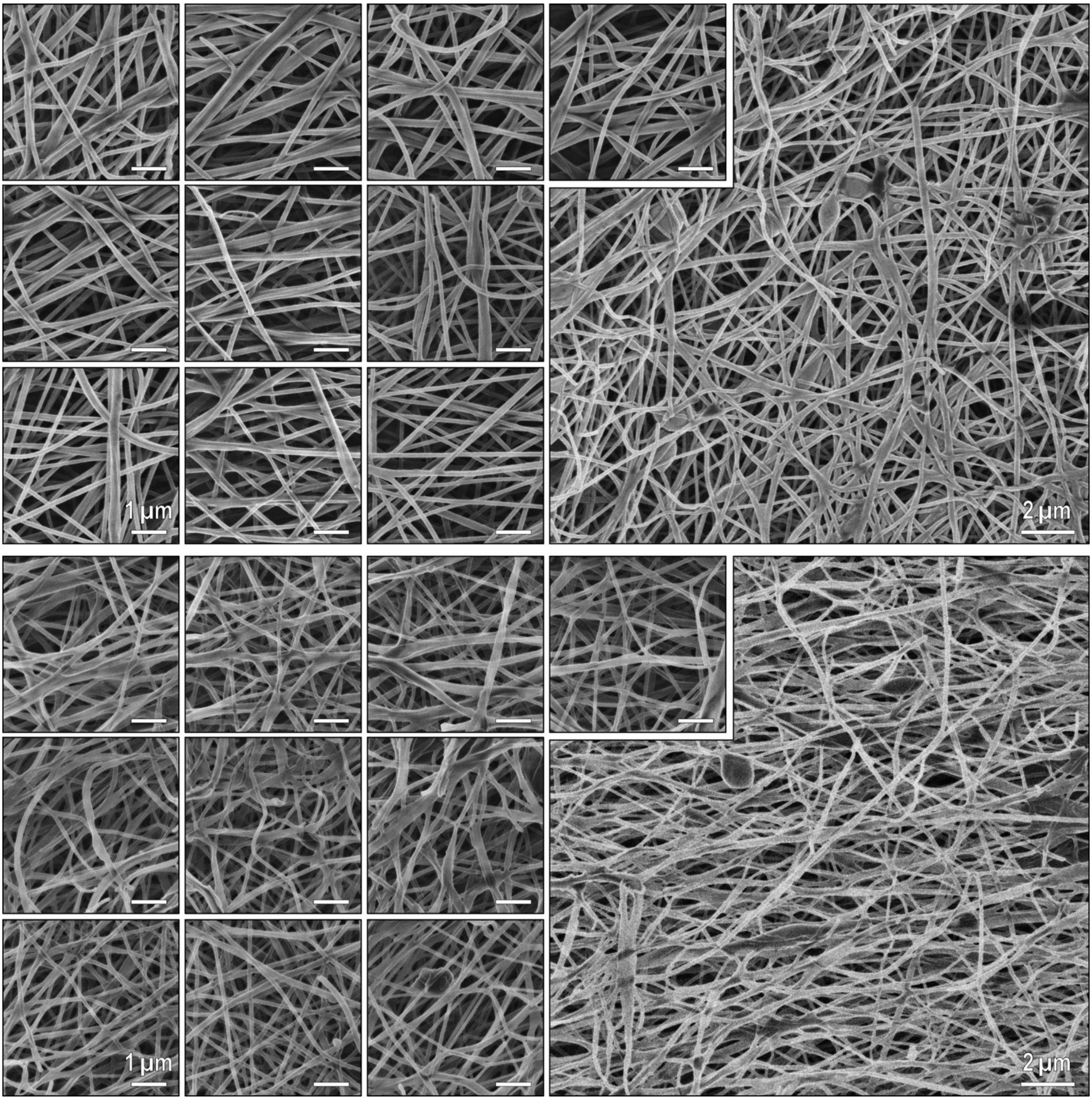

Figure S2: All HIM images that have been analyzed. Gold coated nanofibers are shown in the top half and pristine ones are shown in the bottom half. The images on the left are referred to as *high mag*. and the larger ones on the right are referred to as *low mag*.

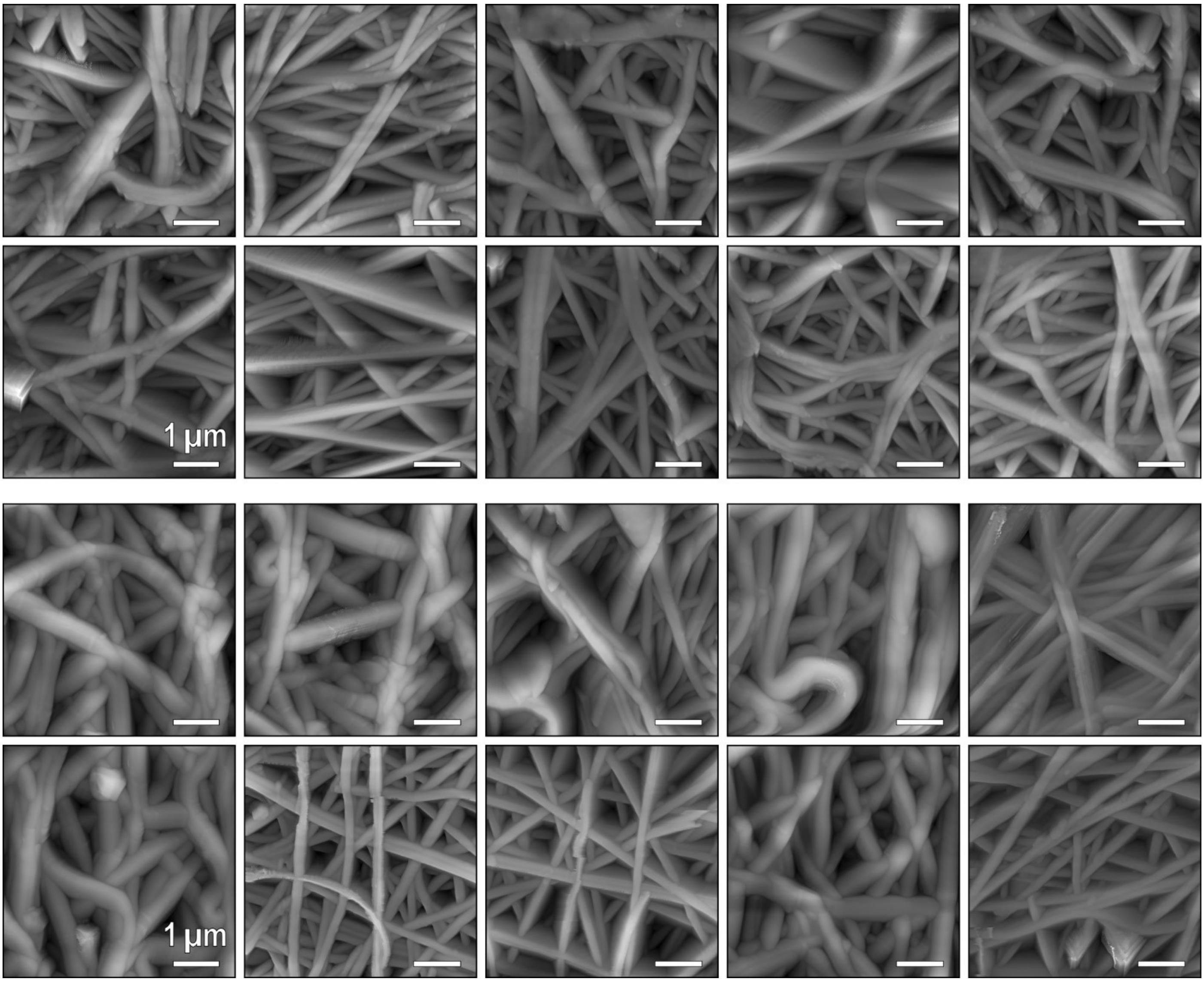

Figure S3: All AFM images that have been analyzed. Gold coated nanofibers are shown in the top half and pristine ones are shown in the bottom half.

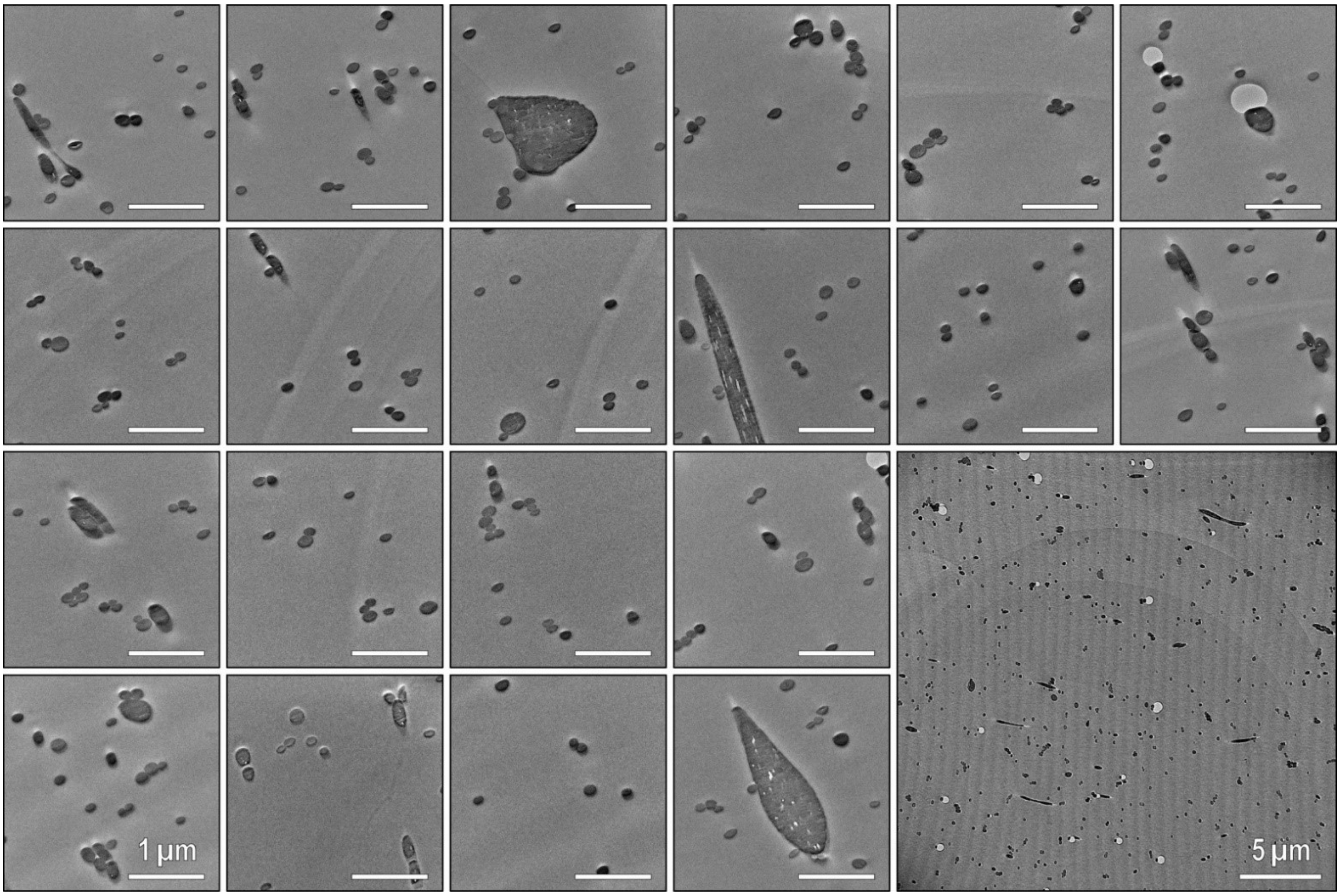

Figure S4: All TEM images that have been analyzed. The larger image on the bottom right is referred to as *low mag*.

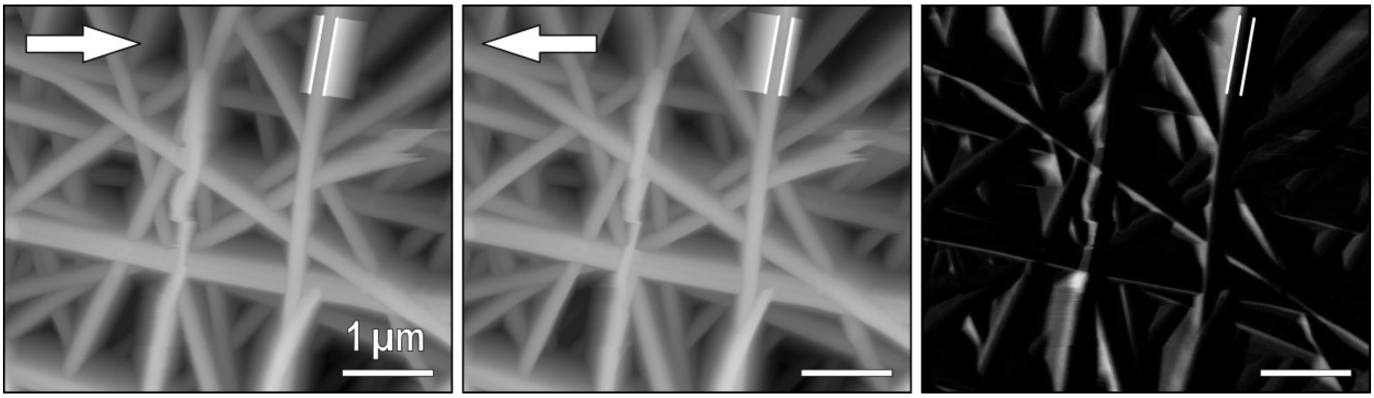

Figure S5: Exemplary AFM images in forward (left) and backward (middle) direction. The right image (contrast increased) is the difference between the forward and backward images illustrating the hysteresis effect. It can be seen that the effect is most pronounced for fibers perpendicular to the scan direction and away from crossing points, i.e. in freestanding regions.

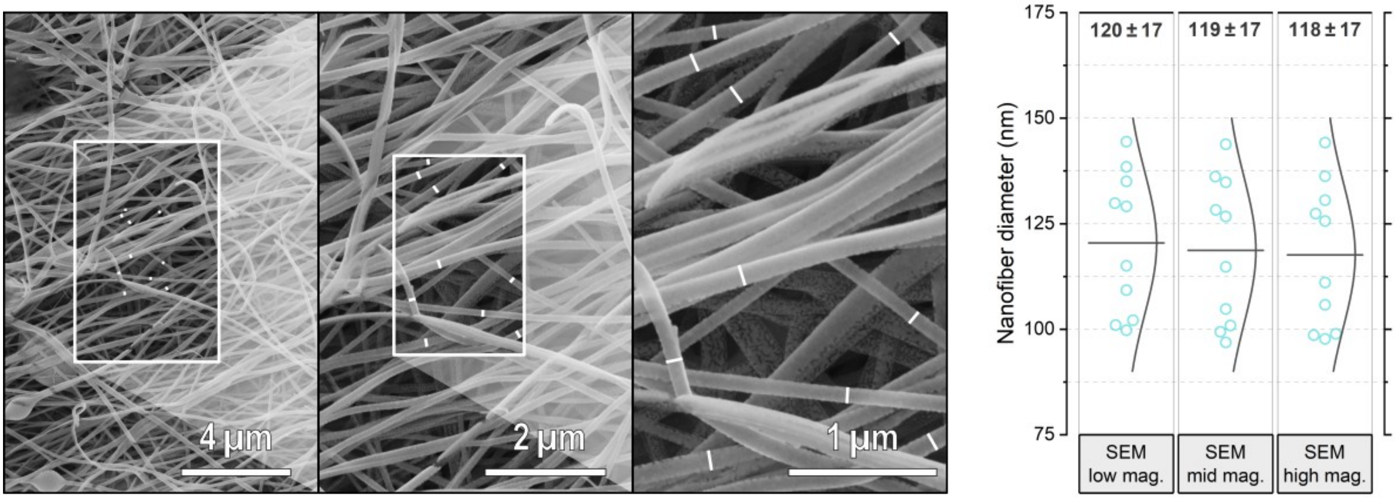

Figure S6: SEM images recorded at different levels of magnification. The nanofiber diameters have been measured by R1 at the same locations indicated by the white lines. No significant difference was found. Note that the mean value is very close to the value given in Fig. 2, however, the STD is much lower (17 nm vs. 33 nm), emphasizing the finding that the diameters appear narrower distributed within single images than across multiple images at different sample regions.

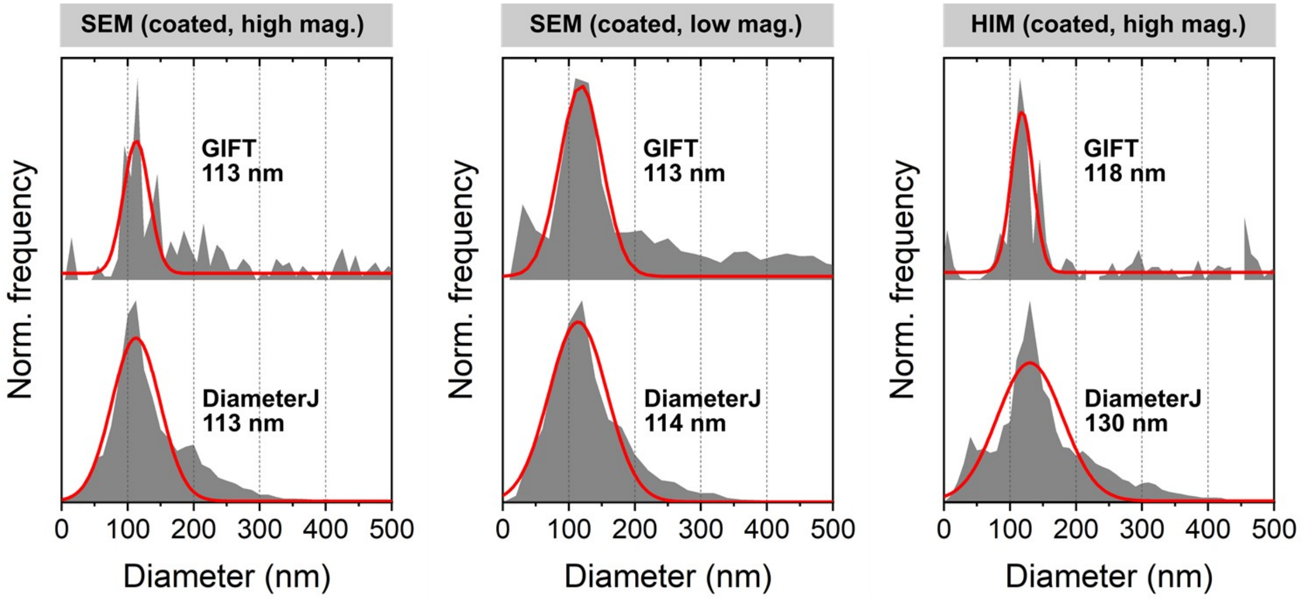

Figure S7: Comparison of diameter distributions generated by GIFT (top) and DiameterJ (bottom). The Gaussian functions are fitted to the primary peak of the combined data sets. The peak maxima are given in the diagrams. In contrast to DiameterJ, the data generated by GIFT showed noise up until 4000 nm, which is not shown here. GIFT gave reasonable results only for half the HIM images of coated nanofibers. Although DiameterJ gave results for all HIM images of coated nanofibers, it was apparent that the overall results are highly unreliable based on both the diameter histogram and the image segmentations.
